# Predictive Modeling of Changes in TBARS in the Intramuscular Lipid Fraction of Raw Ground Beef Enriched with Plant Extracts

**DOI:** 10.3390/antiox10050736

**Published:** 2021-05-07

**Authors:** Anna Kaczmarek, Małgorzata Muzolf-Panek

**Affiliations:** Department of Food Quality and Safety Management, Faculty of Food Science and Nutrition, Poznań University of Life Sciences, Wojska Polskiego 31, 60-637 Poznań, Poland; malgorzata.muzolf-panek@up.poznan.pl

**Keywords:** lipid oxidation, beef, spices, herbs, kinetic models, Arrhenius model, log-logistic model, neural network, temperature effect

## Abstract

The aim of the study was to develop and compare the predictive models of lipid oxidation in minced raw beef meat enriched with selected plant extracts (allspice, basil, bay leaf, black seed, cardamom, caraway, cloves, garlic, nutmeg, onion, oregano, rosemary and thyme) expressed as value changes of TBARS (thiobarbituric acid reactive substances) in various time/temperature conditions. Meat samples were stored at the temperatures of 4, 8, 12, 16 and 20 °C. The value changes of TBARS in samples stored at 12 °C were used as the external validation dataset. Lipid oxidation increased significantly with storage time and temperature. The rate of this increase varied depending on the addition of the plant extract and was the most pronounced in the control sample. The dependence of lipid oxidation on temperature was adequately modeled by the Arrhenius and log-logistic equation with high average *R*^2^ coefficients (≥0.98) calculated for all extracts. Kinetic models and artificial neural networks (ANNs) were used to build the predictive models. The obtained result demonstrates that both kinetic Arrhenius (*R*^2^ = 0.972) and log-logistic (*R*^2^ = 0.938) models as well as ANN (*R*^2^ = 0.935) models can predict changes in TBARS in raw ground beef meat during storage.

## 1. Introduction

Beef consumption has accounted for about 70 million metric tons per year in recent years (2016–2020) worldwide and this type of meat is the third most popular worldwide just after pork and poultry. It is predicted that global beef production and consumption will grow over the next 10 years [1,2] even though a high content of saturated fatty acid (SFA) has led to an unfavorable image by some consumers who associate beef consumption with the risk of chronic diseases [3].

Meat is especially susceptible to quality deteriorations [4]. Lipid oxidation is the main process responsible for the decrease of the sensory and nutritional values of meat. Its extent depends on the content and type of the lipids (including fatty acid composition), the heme pigment content, the presence of other endogenous pro-oxidative and antioxidative agents, the processing methods to which meat is subjected to (such as grinding), the time and temperature conditions during storage, exposure to light and heat and the presence of molecular oxygen [5]. To counteract the negative changes of meat during storage, various technological processes can be implemented including the use of antioxidants.

In recent years there has been an increasing demand for natural food without any artificial preservatives. However, without any food processing ensuring food safety, a constant food supply all over the world and the convenience with which we have now access to all of the products for a proper diet would be impossible. The safety and high quality of food is crucial; thus, in order to meet consumers’ expectations in terms of a “clean label” and additionally enabling the maintenance of the high quality of food during its shelf-life, naturally occurring antioxidants are used as food preservatives [4,6]. Herbs and spices are good sources of antioxidant active compounds [7,8] and since ancient times have been used not only for food seasoning but also for the maintenance of food safety [9]. It was previously shown that some spices and herbs could delay the oxidative and microbial changes of raw ground pork and chicken meat during chilled storage [7,10].

Temperature is one of the most important factors influencing the final quality of stored meat and TBARS have often been used as the index of lipid oxidation in meat and meat products. Thus, to ensure meat safety through the management of food quality during storage it is important to develop models describing and predicting the quality of the product in a wide range of temperatures. Previously, various mathematical models were implemented to describe the quality indices of food during a storage period [11,12,13,14,15,16,17]. The Arrhenius equation was used to predict the temperature dependency of the lipid protein oxidation as well as the microbiological quality of rabbit meat [11,12], pork sausages [16,18], bream fillets [19], minced beef [20] and the lipid oxidation of canola and sesame oils [14] as well as Kilka fish oil [15]. However, there is still a lack of kinetic studies on the lipid oxidation of raw ground beef with extract additions. In addition, according to the knowledge of the authors, artificial neural networks (ANNs) have not been previously used for the description of lipid oxidation in beef. In recent studies, the denaturation of pork meat proteins [21] and the quality of bream fillets [19] were investigated based on ANNs. ANNs were also applied to monitor chicken meat authenticity [22], to distinguish volatile compounds from meat cuts using a GC-MS analysis [23] and to predict the multiple quality of dry-cured ham based on protein degradation [24]. The ANNs, known as “black-box” models, do not make any assumption on the relations between variables and could be applied for the huge data matrix, providing very complex functions including non-linear multiple regressions [25,26]. The main advantage of ANNs is the ability to learn and adapt to the changing experimental conditions and to generalize, which enables the usage of the model to the unlearnt (new) data [21,26].

The Arrhenius equation is often implemented to describe the effect of temperatures but the log-logistic model can be also applied [27]. Both models were successfully applied to modelling quality changes in Songpu mirror carp (*Cyprinus carpio*) during storage at chilled temperatures [28]. However, the application of the log-logistic model in the prediction of meat quality is scarce.

Thus, the aim of this study was to investigate the lipid oxidation of raw ground beef in terms of the values of TBARS during storage at different temperatures and to build the kinetic models as well as ANN models pointing at the prediction of the extent of lipid oxidation in the product. This enables the monitoring and management of the quality changes during the shelf-life of the product. Moreover, for the first time, the predictive models of the oxidative status of raw meat with the addition of plant extracts were constructed and compared.

## 2. Materials and Methods

### 2.1. Materials

Dried allspice, basil, bay leaf, black seed, cardamom, caraway, cloves, garlic, nutmeg, onion, oregano, rosemary and thyme were purchased from a local distributor of herbs and spices (Ciecierzyn, Poland). Beef neck was supplied by a local meat producer (Swarzędz, Poland). The meat was cut and minced by a 5 mm diameter plate. The meat was then immediately transported to the laboratory keeping the temperature value in the range of 4–8 °C during the transport.

### 2.2. Plant Extract Preparation and Characterization

The binary water–ethanol (1:1 *v*/*v*) extracts of spices and herbs were prepared as previously described [7]. The antioxidant activity was measured according to the DPPH method introduced by Sánchez-Moreno et al. [29] with a few modifications [10] and expressed as µmol TE (Trolox equivalent)/g of the dried herb or spice. The total phenolic content was assessed using a Folin–Ciocalteu reagent by the assay of Singleton and Rossi [30]. The final results were presented as the mg of the gallic acid equivalent (GAE) per 1 g of dried herb or spice.

### 2.3. Meat Sample Preparation and Storage

The meat samples with plant extracts were prepared as described by Muzolf-Panek et al. [10]. Briefly, each freeze-dried extract was mixed separately with the meat after dissolving in water (60 mL). The concentration of the spice extract was 0.5% (mass of powdered spice or herb/mass of meat). Fourteen samples were prepared from raw ground beef: one control (meat without extract, only mixed with 60 mL of water) and thirteen treated samples, namely, allspice, basil, bay leaf, black seed, cardamom, caraway, cloves, garlic, nutmeg, onion, oregano, rosemary and thyme. Each sample was then stored at 4, 8 or 12 °C for 13 days and at 16 or 20 °C for 5 days.

### 2.4. Determination of TBARS 

A TBARS index was used to evaluate the degree of lipid oxidation during storage. The presence of TBARS is caused by the second stage of auto-oxidation in which peroxides are oxidized to aldehydes and ketones. The values of TBARS were evaluated based on the method of Mielnik et al. [31] with a few modifications described previously [7]. The values of TBARS were expressed in mg of malondialdehyde (MDA) per kg of meat. In order to universalize the obtained models, percentage changes of the values of TBARS during the storage of meat samples at different temperatures were used for their construction.

### 2.5. Kinetic Analysis

An analysis of the effects of plant extract addition and storage (time and temperature) on the value changes of TBARS was performed by fitting experimental values to kinetic models. Data of TBARS obtained at a constant temperature (4, 8, 12, 16 and 20 °C) were fitted by a conventional first-order model:

(1) $$TBARS=TBARS_{0}\exp\left(kt\right)$$

where *TBARS* is a value of the *TBARS* index (%), *TBARS*_0_ is the initial value (100%) at time 0 and *k* is the food quality rate constant (day^−1^) at a given temperature. The kinetic curves of the reactive substances of *TBARS* were drawn by plotting the changes in the value of *TBARS* (%).

### 2.6. Temperature Dependency

The temperature dependency of the reactive substance formation of TBARS in meat lipids could be assessed using the Arrhenius equation:

(2) $$k=k_{0}\;exp\left(-E_{a}/RT\right)$$

where *k* (day^−1^) represents the formation rate of TBARS, *k*_0_ is the pre-exponential factor, *E_a_* (J/mol) is the activation energy, *R* is the universal gas constant and *T* is the absolute temperature.

The modified logistic Arrhenius equation was given by the equation:

(3) $$lnk=lnk_{0}-E_{a}/RT.$$

An alternative for the Arrhenius equation is a log-logistic relationship [32]:

(4) $$k=m\prime \ln(1+\exp\left(\left[c\left(T-T_{c}\right)\right]\right)$$

where *c* (°C^−1^), *m*′ (-) and *T_c_* (°C^−1^) are empirical fit constants and in many cases it can be assumed that *m*′ = 1. This equation does not need the concept of activation energy.

### 2.7. Artificial Neural Networks (ANNs)

In this study, the STATISTICA Neural Networks simulator was utilized. This tool offers current programming strategies and has many information data analysis instruments that support the generation of the ANNs. The ANNs used storage conditions (time and temperature) and the plant extract addition as the input data for the calculations. The datasets were divided into three subsets in a ratio of 2:1:1. These were a training set (a set of samples used to adjust the network weights), a validation set (a set of samples used to tune the parameters) and a test set (a set of samples used only to assess the performance to new, unseen observations). The Broyden–Fletcher–Goldfarb–Shanno learning algorithm (200 epoch) was used for training multilayer feed-forward connected ANNs and multilayer perceptron (MLP) and radial basis function (RBF) networks were used to search for an appropriate ANN model. Various activation functions in hidden as well as output neurons such as logistic, hyperbolic tangent, exponential, sine, SoftMax and Gaussian were also tested. In the hidden and output layer, each neuron was connected to all of the nodes in the proceeding layer by an associated numerical weight. The weight connecting two neurons regulated the magnitude of the signal that passed between them. To train a neural network, a method of supervised learning was employed and its level was controlled by a validation error in subsequent learning periods. The whole methodology (algorithms and functions) are described on the website of tibco.com (24.03.2021) [33]. The best five out of twenty evaluated networks were retained. The network structure developed for the data of TBARS (%)included an input layer, one hidden layer and an output layer. The input layer was made up of 16 neurons and there were 3–7 neurons in a hidden layer. One neuron in the output layer predicted the values of TBARS (%). The sums of squares and the cross-entropy error function were used during the network training process. The adequacy of the model for the prediction of the values of TBARS was assessed as training performance, validation performance and test performance. The performance was a percentage of the sample in the corresponding dataset (training, validation and test) correctly predicted in the corresponding step (training, validation and test).

### 2.8. Validation and Evaluation of Kinetic and ANN Models

An external validation was performed. The value change of the models of TBARS at 4, 8, 16 and 20 °C were established by combining a kinetic analysis and the Arrhenius equation or a kinetic analysis and the log-logistic equation as well as ANN models. Changes in TBARS at 12 °C were adopted to evaluate the performance of obtained predictive models.

### 2.9. Regression Modeling

To compare the rates (slope of regression equation) of the formation of TBARS in meat samples with different plant extracts within a storage period at a given temperature, a multiple linear regression (MLR) analysis was performed. TBA reactive substances increase exponentially; therefore, a logarithmic transformation was used to linearize this relationship. The general model of the MLR has the following equation:

(5) $$y=\beta_{0}+\beta_{1}\;x_{1}+\beta_{2}\;x_{2}+\cdots +\beta_{k}x_{k}+\varepsilon$$

where *y* is the variable value, *β*_0_ is the intercept, *β*_1*−k*_ is the regression coefficient, *x*_1*−k*_ are the predictors and *ε* is the standard estimation error. The comparisons between the coefficients were performed introducing 13 (*k* − 1) dummy variables as predictors to the regression analysis. The control samples were not coded because this was the category with which all other categories would be compared. The significant differences between the regression coefficients were based on the result of the *t*-test (*p* ≤ 0.05) for the dummy variables.

### 2.10. Statistical Analysis

The measurements of TBARS were run in triplicate and the results were expressed as mean ± standard deviations (SDs). The statistical tests were performed using Statistica 13.3 software (StatSoft, Tulsa, OK, USA). A significance level of *p* = 0.05 was used.

The values of the kinetic parameters were evaluated using a non-linear estimation analysis by a least-squares criterion with a Levenberg–Marquardt algorithm. The goodness of fit of the models was verified based on the determination coefficient (*R*^2^) and the root-mean-square error (RMSE).

## 3. Results and Discussion

### 3.1. Antioxidant Activity and Phenolic Content of Spice Extracts

The antioxidant activity and phenolic content of spice and herb extracts is shown in Table 1 and is discussed in paper [34]. The results of the antioxidant activity and phenolic content in allspice, bay leaf, black seed, caraway, cardamom, clove and nutmeg were previously published [10]. The values of TPC were positively correlated with the DPPH radical scavenging capacity (*r* = 0.98, *p* = 0) and were in agreement with previous observations [7,10]. The highest content of phenolic compounds and the highest antioxidant activity were recorded for clove extract; 167 mg GAE/g and 1443 µM TE/g, respectively. Similar TPC values for clove were obtained by [35,36,37]. However, the antioxidant aqueous ethanol (80%) extract of clove exhibited a slightly higher phenolic content equal to 230 mg GAE/g [38]. Moreover, allspice, thyme, bay leaf, oregano and basil showed both high antioxidant activity and a high phenolic content. The same order was reported by Assefa et al. [37] for an 80% methanol extract of selected spices and herbs. Generally, it is hard to compare the results of the phenolic content and the antioxidant activity of extracts directly with the literature data because various extraction conditions were applied.

### 3.2. Development of Mathematical Models for the Formation of TBARS in Ground Beef Meat

All meat samples were kept under controlled conditions and taken for analysis in appropriate time intervals to allow for the efficient kinetic analysis of secondary lipid oxidation in products measured using the index of TBARS. The highest regression coefficients values were obtained for the logarithmic plot of the value vs. time of TBARS. Therefore, the first-order reaction model was applied (Equation (1)). The effect of the temperature was included in the mathematical models using the Arrhenius equation (Equation (3)) and the log-logistic (Equation (4)) equations. The predictive models were obtained by integrating Equations (1) and (3) and Equations (1) and (4).

### 3.3. Arrhenius Models

With the first reaction order and corresponding rate constant derived from chemical kinetics, the parameters in the Arrhenius models (Equation (3)) were calculated by linear regression (lnk vs. 1/T). The results are presented in Table 2. The Arrhenius models described adequately the temperature dependency with high average values of the determination coefficient calculated for all extracts equaling 0.98.

The highest *R*^2^ value was noted for the Arrhenius parameters obtained based on the changes in TBARS in the control sample and the beef sample with the cardamom extract addition (*R*^2^ = 0.997) whereas the lowest was in meat samples enriched with caraway extract (*R*^2^ = 0.887). The *E_a_* values for the formation of TBARS varied from 27,250 J/mol for the caraway-treated sample to 131,842 J/mol for the black seed-treated sample (Table 2). Therefore, the samples can be ordered from the most sensitive to temperature to the least sensitive to temperature in the following order: black seed > clove > allspice > oregano > rosemary ≥ basil > bay leaf > onion > cardamom ≈ thyme > garlic > nutmeg ≥ control > caraway. This could suggest that the black seed and clove addition to the meat made the reaction rates more susceptible to the temperature whereas in the caraway-treated samples, the lipid oxidation rates were less temperature dependent with a lower activation energy in comparison with the control sample. However, the *k*_0_ values representing how fast the oxidation occurred were the lowest for the caraway-treated beef samples and the highest were in the black seed-treated sample, which meant that the rate of the changes in TBARS in the beef sample with the caraway addition was relatively slow with the storage period. The opposite effect was observed for the black seed addition to beef. Clove was previously reported as the most antioxidant active extract in pork and chicken meat samples [7,10] and caraway in pork meat [10] but black seed was an effective extract in the maintenance the oxidative stability of chicken meat [10]. This proved that the lipid oxidation is a very complex process affected significantly by the temperature during storage and, to compare the effects of extract additions in meat with lipid oxidation, a broad study is needed. Thus, to limit a large number of experimental measurements necessary for the assessment of meat quality, a predictive approach using kinetic models could be applied.

The Arrhenius model of changes in TBARS in ground beef meat with the addition of various plant extracts was given in the equation:

(6) $$TBARS=TBARS_{0}\exp\left(k_{0}\;\exp\left(-E_{a}/RT\right)\;t\right)$$

where *TBARS* is the value of the index of *TBARS* (%), *TBARS*_0_ is the initial value (100%) at time 0, *k*_0_ represents the formation rate of TBARS, *E_a_* is the activation energy, *R* is the universal gas constant, *T* is the absolute temperature and *t* is the storage time.

The goodness of fit of the Arrhenius models are given in Table 3. The average values of the adjusted *R*^2^ between the observed and the predicted values of TBARS were in the range from 0.780 to 0.990. The highest average value of the determination coefficient was noted for the control sample whereas the lowest was for the rosemary-treated sample. The sum of *R*^2^ was also the highest for the control sample in the tested temperatures (*R*^2^ = 3.96) than for the extract-treated samples (Table 3).

### 3.4. Log-Logistic Model

An alternative to the Arrhenius model is the log-logistic model (Equation (4)). The parameters of the obtained models are shown in Table 2. The high average regression coefficients (*R*^2^ = 0.983) indicated that the log-logistic temperature dependency well described this relation in tested samples. The highest *R*^2^ value was observed for the log-logistic model obtained based on the changes of TBARS in the meat sample with the oregano extract addition (*R*^2^ = 0.999) while the lowest was in the meat sample enriched with rosemary extract (*R*^2^ = 0.883).

The log-logistic model of the changes of TBARS in ground beef meat with various plant extract additions was given in the equation:

(7) $$TBARS=TBARS_{0}\exp\left(\ln\left(1+\exp\left(c\left(T-T_{C}\right)\right)\right)t\right)$$

where *TBARS* is the value of the index of *TBARS* (%), *TBARS*_0_ is the initial value (100%) at time 0, *c* (°C^−1^) and *T_c_* (°C^−1^) are empirical fit constants and *t* is the storage time.

The goodness of fit of the log-logistic models are presented in Table 3. The average values of the *R*^2^ coefficient for the observed and the predicted values of TBARS were in the range of 0.800 to 0.991. The highest value of the determination coefficient was noted for the oregano-treated sample whereas the lowest was for the rosemary-treated sample. The sum of *R*^2^ was also higher for the oregano sample (3.96) in the tested temperatures than for the other samples in the tested temperature range (Table 3). The log-logistic models showed a similar goodness of fit to the Arrhenius models with the average sum of *R*^2^ values equaling 3.72 and 3.73, respectively, and the average *R*^2^ values equaling 0.929 and 0.932, respectively.

### 3.5. ANN Models

The best five ANN-MLP networks are presented in Table 4. In the neural network obtained for the values of TBARS, the Tanh and exponential functions were used in the hidden layer while the exponential and linear functions were used in the output layer. The number of neurons in the hidden layer varied from 3 to 10. The goodness of fit of all selected networks was very high. The best network was MLP 16-7-1 with the highest adjusted determination coefficient (*R*^2^ = 0.9929) and the lowest were the RMSE (16.10) values.

### 3.6. Validation and Evaluation of Quality Prediction Models

**Figure 1:**
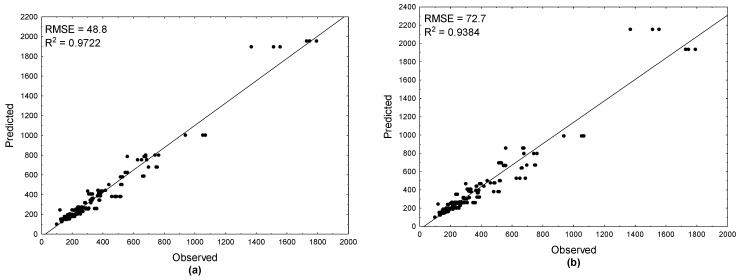

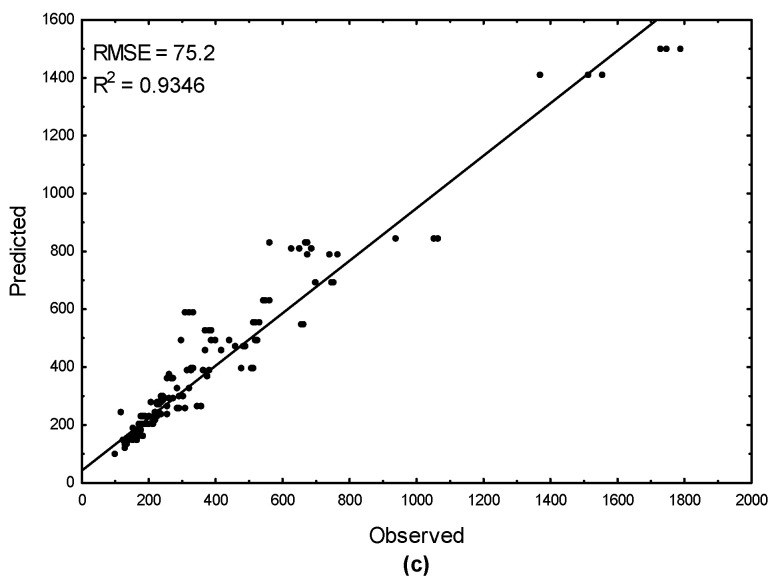
Predictability of the (**a**) Arrhenius model; (**b**) log-logistic model; (**c**) ANN model for the value changes of TBARS in beef samples enriched with plant extracts stored at 12 °C. The solid line represents a perfect match between the experimental and the predicted values.

The validation of TBARS calculated through predictive models was measured by the changes of TBARS of samples at 12 °C. The value changes of TBARS during the storage of meat samples predicted using these three models were plotted against the observed values (Figure 1). The plot for the ANN model shown in Figure 1c was a combination of all five best networks. The scatter plots revealed a high order of linearity, which was confirmed by high adjusted regression coefficients (0.9346–0.9722) and low RSME values. The best prediction ability was noted for the Arrhenius model (*R*^2^ = 0.9722, RMSE = 48.8). The worst forecasting ability with the highest RMSE value (75) was reported for the ANN model. This was explained by the fact that in the network model, the type of plant extract was introduced as an additional attribute predictor. The reason for the high RMSE values for the log-logistic (RMSE = 72) model validation was that the sum of the determination coefficients calculated for the model fitted to the data for TBARS; the log-logistic model was slightly lower (52.29) than for the Arrhenius model (52.43) In general, the models obtained tended to overestimate the values of TBARS especially at higher temperatures. Even though the models obtained overestimated the predicted values, they could be used for the safe prediction of these index values in the lower temperature range. The obtained models may be helpful in estimating the shelf-life of meat.

### 3.7. Regression Modeling Using MLR

To assess the influence of time, temperature and the addition of plant extracts on the value of the increase in lipid fraction of beef TBARS, an MLR was performed. The results of the regression analysis are shown in Table 5. The higher the absolute value of the regression coefficients were, the higher the differences between the control and the treated samples; thus, the slower the oxidation of lipids. The multiple regression analysis was statistically significant with a *p*-value < 0.01 apart from the sample with the onion addition. According to the regression coefficient values, all plant extracts inhibited lipid oxidation changes in beef meat (negative values of coefficients). Based on the result of this analysis, it could be concluded that generally spices such as allspice, bay leaf and clove with the highest antioxidant activity and phenolic content decreased the oxidative changes the most. Bay leaf extract possessed the best ability to inhibit the oxidation process in meat samples with the highest slope value (−0.8682). Surprisingly, in this study, cardamom, which exhibited a very low antioxidant activity, showed a regression coefficient similar to oregano, which suggested a similar inhibitory effect against lipid oxidation in beef meat. Previously, it was reported that cardamom significantly increased the oxidative stability of lipids in raw pork even at a higher extent than allspice or bay leaf [10]. Allspice and clove were shown to inhibit significantly lipid oxidation in chicken meat whereas clove was the most antioxidant active extract in pork meat [7,10].

## 4. Conclusions

This study explores the effect of temperature and the antioxidant properties of selected culinary spices and herbs on the secondary lipid oxidation product changes measured by the index of TBARS in raw ground beef meat stored under different temperatures. The models employed could be used for the prediction of oxidative changes in the intramuscular fat fraction of beef. The validation of the models enabled us to conclude that the Arrhenius model showed a slightly better accuracy to the experimental data than the model based on the log-logistic equation or ANN models. This study demonstrated the potential usefulness of the models for a realistic prediction of the changes in TBARS in raw beef meat during storage. Such predictive models allow the monitoring of oxidative changes in ground meat under different time and temperature conditions. This knowledge is very useful for designing food products and predicting their shelf-life. Moreover, the effectiveness of various spices in the raw beef meat system was compared based on MLR and showed that clove, allspice and bay leaf were the most potent antioxidant active extracts. However, it is important to stress that meat is a very complex system and, according to the research, there is no direct correlation between the antioxidant activity of the spice itself and its antioxidant effectiveness in the product. Therefore, it is necessary to test their efficiency in particular food products.

## Figures and Tables

**Table 1:** Antioxidant activity and phenolic compound content of ethanol in water (1/1 *v*/*v*) of extracts.

Extracts	DPPH µM TE/g	TPC mg GAE g/DW
Allspice *	555 ± 24 ^g^	31.61 ± 0.81 ^e^
Basil	134.7 ± 2.3 ^c^	14.81 ± 0.35 ^bc^
Bay leaf *	231.9 ± 1.5 ^e^	22.56 ± 0.16 ^cd^
Black seed *	7.59 ± 0.84 ^a^	2.46 ± 0.61 ^a^
Cardamom *	5.45 ± 0.35 ^a^	1.24 ± 0.01 ^a^
Caraway *	20.2 ± 0.6 ^a^	2.39 ± 0.14 ^a^
Clove *	1443 ± 1 ^h^	167.2 ± 9.3 ^f^
Garlic	14.8 ± 1.6 ^a^	3.6 ± 0.05 ^a^
Nutmeg *	22.22 ± 0.15 ^ab^	3.89 ± 0.14 ^a^
Onion	5.74 ± 0.28 ^a^	7.05 ± 0.58 ^ab^
Oregano	171.6 ± 5.8 ^d^	20.7 ± 0.1 ^cd^
Rosemary	50.4 ± 3.6 ^b^	4.66 ± 0.36 ^a^
Thyme	278.3 ± 16.2 ^f^	23.5 ± 0.6 ^d^

All values are mean ± SD of the three replicates. (*) data from [10]. TPC = total polyphenol content. ^(a–h)^ means with the same superscript within the same column are not different (*p* > 0.05).

**Table 2:** Parameters of the Arrhenius model and log-logistic model for the value changes of the TBARS of ground beef with plant extracts during storage at different temperatures.

Extracts	Temperature (K)	*k*	Arrhenius Model			Log-Logistic Model		
			*R* ^2^	*E_a_* (J/mol)	*k*_0_ (d^−1^)	*R* ^2^	C (°C^−1^)	*T_c_* (°C^−1^)
Control	277	0.14672 ± 0.00218	0.9972 ± 0.0005	60,292 ± 863	1.7 × 10^11^ ± 3.8 × 10^10^	0.9955 ± 0.0010	0.1094 ± 0.0015	21.73 ± 0.08
	281	0.20124 ± 0.00371						
	289	0.40969 ± 0.00084						
	293	0.61344 ± 0.00387						
Allspice	277	0.01624 ± 0.00039	0.9794 ± 0.0011	118,544 ± 1355	4.7 × 10^20^ ± 2.8 × 10^20^	0.9973 ± 0.0009	0.1615 ± 0.0027	26.53 ± 0.13
	281	0.05191 ± 0.00096						
	289	0.17147 ± 0.00998						
	293	0.29703 ± 0.00748						
Basil	277	0.05460 ± 0.00152	0.9896 ± 0.0018	94,708 ± 168	3.9 × 10^16^ ± 2.6 × 10^15^	0.9786 ± 0.0046	0.1357 ± 0.0023	23.36 ± 0.25
	281	0.09710 ± 0.00032						
	289	0.35292 ± 0.00162						
	293	0.47148 ± 0.01188						
Bay leaf	277	0.02326 ± 0.00205	0.9718 ± 0.0121	88,610 ± 4357	3.37 × 10^15^ ± 4.7 × 10^15^	0.9779 ± 0.0176	0.1495 ± 0.0009	29.48 ± 0.52
	281	0.05404 ± 0.00100						
	289	0.11087 ± 0.01111						
	293	0.22333 ± 0.00295						
Black seed	277	0.02269 ± 0.00135	0.9857 ± 0.0025	131,842 ± 1783	2.2 × 10^23^ ± 1.3 × 10^23^	0.9971 ± 0.0015	0.1827 ± 0.0035	21.74 ± 0.06
	281	0.07358 ± 0.00136						
	289	0.31358 ± 0.01153						
	293	0.54074 ± 0.00553						
Cardamom	277	0.07303 ± 0.00326	0.9972 ± 0.0006	76,564 ± 935	2.1 × 10^13^ ± 7.1 × 10^12^	0.9966v ± 0.0015	0.1288 ± 0.0013	24.13 ± 0.25
	281	0.12740 ± 0.00235						
	289	0.28783 ± 0.00216						
	293	0.46826 ± 0.01179						
Caraway	277	0.09533 ± 0.00176	0.8868 ± 0.0228	27,250 ± 140	1.4 × 10^4^ ± 8.3 × 10^2^	0.8832 ± 0.0222	0.0442 ± 0.0006	54.87 ± 0.12
	281	0.11328 ± 0.00406						
	289	0.14559 ± 0.00501						
	293	0.20404 ± 0.00165						
Clove	277	0.06834 ± 0,00007	0.9891 ± 0.0099	−122,721 ± 646	3.8 × 10^8^ ± 1.5 × 10^8^	0.9867 ± 0.0145	0.1514 ± 0.0034	57.87 ± 0.12
	281	0.08858 ± 0.00006						
	289	0.15796 ± 0.00925						
	293	0.23645 ± 0.00026						
Garlic	277	0.11126 ± 0.00131	0.9941 ± 0.0010	63,945 ± 821	1.2 × 10^11^ ± 3.9 × 10^10^	0.9963 ± 0.0031	0.1121 ± 0.0008	24.11 ± 0.10
	281	0.14729 ± 0.00165						
	289	0.33049 ± 0.01478						
	293	0.49409 ± 0.00183						
Nutmeg	277	0.06435 ± 0.00119	0.9965 ± 0.0035	61,056 ± 898	2.2 × 10^10^ ± 9.9 × 10^9^	0.9929 ± 0.0073	0.0932 ± 0.0008	32.57 ± 0.41
	281	0.09282 ± 0.00171						
	289	0.20392 ± 0.01146						
	293	0.26457 ± 0.00666						
Onion	277	0.11100 ± 0.00017	0.9979 ± 0.0003	79,215 ± 406	8.9 × 10^13^ ± 1.6 × 10^13^	0.9948 ± 0.0024	0.1433 ± 0.0019	20.09 ± 0.25
	281	0.16893 ± 0.00311						
	289	0.53441± 0.00765						
	293	0.69916 ± 0.01761						
Oregano	277	0.04735 ± 0.00112	0.9963 ± 0.0003	105,249 ± 177	3.5 × 10^18^ ± 5.7 × 10^17^	0.9996 ± 0.0001	0.1695 ± 0.0007	21.24 ± 0.21
	281	0.10165 ± 0.00196						
	289	0.34245 ± 0.00984						
	293	0.59431 ± 0.01997						
Rosemary	277	0.05135 ± 0.00103	0.9495 ±0.0056	95,558 ± 1110	6.9 × 10^16^ ± 3.3 × 10^16^	0.9957 ± 0.0016	0.1024 ± 0.0018	28.75 ± 0.57
	281	0.14071 ± 0.00083						
	289	0.43025 ± 0.01579						
	293	0.49277 ± 0.00029						
Thyme	277	0.06746 ± 0.00031	0.9916 ± 0.0004	76,348 ± 195	1.7 × 10^13^ ± 1.4 × 10^12^	0.9840 ± 0.0004	0.1123 ± 0.0005	26.09 ± 0.07
	281	0.11290 ± 0.00054						
	289	0.30439 ± 0.00057						
	293	0.39286 ± 0.00182						

**Table 3:** The goodness of fit of the Arrhenius and log-logistic models of the changes of TBARS in ground beef meat with the addition of various plants extracts during storage at different temperatures.

Extract	Temperature (K)	Model					
		Arrhenius			Log-Logistic		
		*R* ^2^	RMSE	Σ*R*^2^	*R* ^2^	RMSE	Σ*R*^2^
Control	277	0.9891 ± 0.0097	17.74 ± 10.40	3.96	0.9551 ± 0.0243	38.28 ± 12.75	3.94
	281	0.9866 ± 0.0183	34.69 ± 26.22		0.9961 ± 0.0017	22.48 ± 5.07	
	289	0.9968 ± 0.0019	10.27 ± 2.61		0.9932 ± 0.0043	14.76 ± 4.00	
	293	0.9882 ± 0.0111	39.58 ± 22.61		0.9931 ± 0.0054	31.18 ± 13.95	
Allspice	277	0.9879 ± 0.0027	3.95 ± 0.28	3.71	0.9289 ± 0.0122	9.60 ± 0.44	3.88
	281	0.7939 ± 0.0325	13.51 ± 1.46		0.9815 ± 0.0110	3.92 ± 1.47	
	289	0.9729 ± 0.0176	5.42 ± 1.31		0.9695 ± 0.0239	5.64 ± 1.75	
	293	0.9521 ± 0.0278	20.36 ± 4.92		0.9972 ± 0.0028	4.71 ± 2.11	
Basil	277	0.9982 ± 0.0016	2.80 ± 2.14	3.75	0.9327 ± 0.0231	20.41 ± 2.75	3.48
	281	0.9924 ± 0.0055	6.52 ± 2.39		0.6547 ± 0.0476	45.97 ± 2.52	
	289	0.8800 ± 0.0226	38.63 ± 4.02		0.9126 ± 0.0198	32.93 ± 4.07	
	293	0.8760 ± 0.0613	76.42 ± 14.17		0.9797 ± 0.0205	28.62 ± 13.51	
Bay leaf	277	0.9633 ± 0.0180	4.14 ± 0.77	3.60	0.9752 ± 0.0127	3.44 ± 1.05	3.48
	281	0.8660 ± 0.0297	11.77 ± 1.55		0.7189 ± 0.0404	17.09 ± 1.55	
	289	0.8534 ± 0.0388	8.66 ± 0.51		0.8711 ± 0.0321	8.13 ± 0.46	
	293	0.9128 ± 0.0191	17.74 ± 2.01		0.9162 ± 0.0242	17.32 ± 2.21	
Black seed	277	0.9975 ± 0.0015	4.87 ± 1.41	3.78	0.9727 ± 0.0071	16.51 ± 1.55	3.93
	281	0.9189 ± 0.0137	19.81 ± 2.56		0.9885 ± 0.0093	7.00 ± 2.51	
	289	0.9679 ± 0.0160	15.3 ± 4.86		0.9779 ± 0.0071	12.74 ± 1.23	
	293	0.8924 ± 0.0268	112.71 ± 11.14		0.9958 ± 0.0033	21.26 ± 9.39	
Cardamon	277	0.8798 ± 0.0162	30.81 ± 1.96	3.70	0.8847 ± 0.0144	30.21 ± 2.45	3.68
	281	0.9756 ± 0.0151	19.37 ± 7.69		0.9566 ± 0.0204	26.35 ± 7.68	
	289	0.8504 ± 0.0342	36.98 ± 3.20		0.8431 ± 0.0356	37.88 ± 3.25	
	293	0.9951 ± 0.0030	14.76 ± 5.77		0.9954 ± 0.0025	14.54 ± 4.85	
Caraway	277	0.9790 ± 0.0179	9.97 ± 3.94	3.84	0.9810 ± 0.0170	9.43 ± 3.94	3.84
	281	0.9200 ± 0.0317	43.78 ± 10.93		0.9166 ± 0.0323	44.71 ± 10.94	
	289	0.9539 ± 0.0340	13.32 ± 5.11		0.9523 ± 0.0346	13.58 ± 5.09	
	293	0.9859 ± 0.0097	6.56 ± 2.41		0.9890 ± 0.0081	5.75 ± 2.29	
Clove	277	0.9892 ± 0.0110	4.18 ± 4.18	3.59	0.8593 ± 0.0729	16.99 ± 3.85	3.59
	281	0.9736 ± 0.0151	5.61 ± 5.61		0.8637 ± 0.2407	36.18 ± 1.10	
	289	0.8047 ± 0.0858	29.75 ± 29.75		0.8855 ± 0.0696	22.55 ± 8.93	
	293	0.8118 ± 0.0647	55.66 ± 55.66		0.9811 ± 0.0169	16.58 ± 6.84	
Garlic	277	0.9895 ± 0.0071	11.03 ± 4.32	3.94	0.9547 ± 0.0141	23.54 ± 4.62	3.92
	281	0.9794 ± 0.0022	25.90 ± 1.01		0.9865 ± 0.0095	19.80 ± 9.07	
	289	0.9887 ± 0.0121	10.15 ± 7.09		0.9869 ± 0.0179	10.22 ± 8.44	
	293	0.9835 ± 0.0071	30.07 ± 7.28		0.9916 ± 0.0038	21.44 ± 5.34	
Nutmeg	277	0.9985 ± 0.0011	1.61 ± 0.54	3.92	0.9887 ± 0.0111	4.24 ± 1.93	3.91
	281	0.9973 ± 0.0041	2.92 ± 2.78		0.9893 ± 0.0120	6.67 ± 3.80	
	289	0.9394 ± 0.0780	9.92 ± 9.13		0.9389 ± 0.0786	9.98 ± 9.15	
	293	0.9885 ± 0.0130	6.39 ± 3.63		0.9938 ± 0.0080	4.49 ± 3.10	
Onion	277	0.9991 ± 0.0015	3.81 ± 4.11	3.94	0.9914 ± 0.0035	16.63 ± 4.65	3.88
	281	0.9800 ± 0.0150	34.22 ± 18.17		0.9298 ± 0.0293	67.67 ± 18.14	
	289	0.9784 ± 0.0169	24.16 ± 9.79		0.9654 ± 0.0274	31.69 ± 10.37	
	293	0.9902 ± 0.0087	54.23 ± 39.39		0.9924 ± 0.0058	50.81 ± 26.39	
Oregano	277	0.9973 ± 0.0012	6.02 ± 0.86	3.93	0.9954 ± 0.0017	7.85 ± 0.87	3.96
	281	0.9533 ± 0.0183	20.67 ± 5.17		0.9794 ± 0.0123	13.45 ± 5.18	
	289	0.9941 ± 0.0047	7.93 ± 3.46		0.9940 ± 0.0047	7.69 ± 3.21	
	293	0.9809 ± 0.0210	49.52 ± 26.00		0.9956 ± 0.0030	25.48 ± 7.38	
Rosemary	277	0.9857 ± 0.0004	14.76 ± 0.91	3.12	0.6662 ± 0.0243	71.17 ± 0.91	3.20
	281	0.8539 ± 0.0097	54.86 ± 2.99		0.7937 ± 0.0259	65.03 ± 2.70	
	289	0.7551 ± 0.0647	83.53 ± 17.86		0.7868 ± 0.0627	77.89 ± 17.81	
	293	0.5259 ± 0.0945	178.42 ± 11.42		0.9535 ± 0.0116	55.74 ± 5.14	
Thyme	277	0.9956 ± 0.0024	4.25 ± 1.10	3.67	0.9286 ± 0.0145	17.38 ± 1.61	3.60
	281	0.9891 ± 0.0058	9.87 ± 2.82		0.8967 ± 0.0163	31.17 ± 2.45	
	289	0.8116 ± 0.0108	38.20 ± 1.13		0.8212 ± 0.0106	37.22 ± 1.14	
	293	0.8715 ± 0.0145	48.11 ± 2.28		0.9512 ± 0.0070	29.63 ± 1.83	

**Table 4:** ANN model parameters for changes in TBARS in ground beef meat enriched with plant extracts stored at different temperatures.

Net Parameters	Net Structure				
	MLP 16-5-1	MLP 16-10-1	MLP 16-4-1	MLP 16-7-1	MLP 16-3-1
Training accuracy	0.993	0.995	0.996	0.997	0.991
Test accuracy	0.992	0.990	0.996	0.996	0.990
Validation accuracy	0.992	0.994	0.996	0.995	0.991
Training error	236.9	194.1	131.6	108.7	311.2
Test error	342.6	371.1	196.2	177.2	374.5
Validation error	322.0	253.9	215.4	229.9	366.6
Training algorithm	BFGS 148	BFGS 284	BFGS 215	BFGS 136	BFGS 203
Error function	SOS	SOS	SOS	SOS	SOS
Hidden activation	Exponential	Tanh	Tanh	Tanh	Exponential
Output activation	Exponential	Linear	Exponential	Exponential	Exponential
*R* ^2^	0.9857	0.9887	0.9921	0.9929	0.9826
RMSE	22.82	20.12	16.91	16.10	25.34

**Table 5:** The results of the multiple linear regression analysis (MLR).

Independent Variables and Intercept	Regression Coefficients	*p*-Values
Bay leaf	−0.8682	1.83 × 10^−51^
Allspice	−0.8151	4.66 × 10^−46^
Clove	−0.7765	2.87 × 10^−42^
Nutmeg	−0.6382	8.34 × 10^−30^
Caraway	−0.5884	8.91 × 10^−26^
Rosemary	−0.5839	2.01 × 10^−25^
Black seed	−0.5551	3.15 × 10^−23^
Basil	−0.4808	5.42 × 10^−18^
Thyme	−0.4591	1.39 × 10^−16^
Cardamon	−0.4361	3.76 × 10^−15^
Oregano	−0.434	5.07 × 10^−15^
Garlic	−0.2362	1.71 × 10^−5^
Onion	−0.0385	4.81 × 10^−1^
Temperature	0.0616	1.82 × 10^−146^
Time	0.1356	1.23 × 10^−264^
Intercept	4.4563	0

## Data Availability

The data that support the findings of this study are published in Mendeley datasets at Kaczmarek, Anna (2021), “TBARS_BEEF”, Mendeley Data, V1, doi:10.17632/cs942c8rw3.1.
